# The Role of Oral Biomarkers in the Assessment of Noncommunicable Diseases

**DOI:** 10.3390/diagnostics15010078

**Published:** 2024-12-31

**Authors:** Gustavo Sáenz-Ravello, Marcela Hernández, Mauricio Baeza, Patricia Hernández-Ríos

**Affiliations:** 1 Centro de Epidemiologia y Vigilancia de las Enfermedades Orales (CEVEO), Faculty of Dentistry, University of Chile, Santiago 9170022, Chile; gustavo.saenz@odontologia.uchile.cl (G.S.-R.); mbaeza.paredes@odontologia.uchile.cl (M.B.); 2 Laboratory of Periodontal Biology, Faculty of Dentistry, University of Chile, Santiago 9170022, Chile; mhernandezrios@odontologia.uchile.cl; 3 Department of Pathology and Oral Medicine, Faculty of Dentistry, University of Chile, Santiago 9170022, Chile; 4 Department of Conservative Dentistry, Faculty of Dentistry, University of Chile, Santiago 9170022, Chile

**Keywords:** oral biomarkers, systemic diseases, early diagnosis, personalized medicine

## Abstract

**Background/Objectives**: Oral biomarkers have gained attention as non-invasive tools for assessing systemic diseases due to their potential to reflect physiological and pathological conditions. This review aims to explore the role of oral biomarkers in diagnosing and monitoring systemic diseases, emphasizing their diagnostic relevance and predictive capabilities in clinical practice. **Methods**: This narrative review synthesizes the current literature on biochemical, immunological, genetic, and microbiological oral biomarkers, with a focus on their sources, types, and clinical applications. Key studies were analyzed to identify associations between oral biomarkers and systemic diseases such as cardiovascular diseases, type 2 diabetes mellitus, autoimmune disorders, and cancers. **Results**: Oral fluids, including saliva and gingival crevicular fluid, contain diverse biomarkers such as matrix metalloproteinases, cytokines, and genetic indicators. These markers have demonstrated potential in diagnosing and monitoring systemic conditions. Among others, elevated levels of salivary glucose and inflammatory cytokines correlate with diabetes progression, while vascular endothelial growth factor (VEGF) and salivary C-reactive protein might be applicable as indicators for periodontal disease and cardiovascular risk. Additionally, salivary biomarkers like amyloid-beta and tau are promising in detecting neurodegenerative disorders. **Conclusions**: Oral biomarkers might represent a transformative and point-of-care approach to the early management of systemic diseases; however, challenges in measurement variability, standardization, and validation remain.

## 1. Introduction

Noncommunicable diseases (NCDs) represent a relevant public health issue. They encompass a considerable health burden, leading to a decline in the overall well-being and quality of life of the population, highly associated with social determinants of health and lifestyle [1,2]. Additionally, NCDs impose adverse socioeconomic consequences to individuals and the healthcare system, acting as a pressing issue in the public health agenda [3]. In this sense, the World Health Organization (WHO) states that to strengthen health systems, it is fundamental to provide NCD services—early detection, screening, and treatment—through a radical reorientation towards primary healthcare which can provide early detection and timely treatment to tackle this global problem [4]. Indeed, NCDs are a barrier to achieving various Sustainable Development Goals (SDGs), such as SDG 1 (No poverty), SDG 2 (Zero hunger), SDG 4 (Quality education), SDG 5 (Gender equality), and SDG 10 (Reduced inequalities). Productivity gains from preventing and managing NCDs contribute to SDG 8 (Decent work and economic growth) [5]. Solving this problem not only requires prevention, but it also needs early detection and intervention.

Among the most common noncommunicable oral diseases, periodontitis affects about 1 billion people, with an age-standardized prevalence of 12.5% globally [6]. Periodontitis is a chronic multifactorial inflammatory disease initiated by a dysbiotic biofilm that leads to the progressive destruction of the tooth-supporting apparatus and eventually tooth loss [5]. Its main signs encompass alveolar bone resorption, clinical attachment loss (CAL), periodontal pocketing, and gingival bleeding [7]. While the associated host–bacterial interactions may trigger local inflammatory responses that account for the release of histolytic enzymes, host tissue components, reactive oxygen species (ROS), cytokines, and other immune mediators, the translocation of bacterial products or inflammatory mediators from periodontal sites to the systemic circulation might trigger and perpetuate low-grade systemic inflammation that may link periodontal and systemic diseases [8].

Until now, periodontitis has been associated with more than 50 different systemic diseases, including several NCDs like diabetes mellitus, cardiovascular diseases, neurodegenerative conditions, chronic kidney disease, metabolic syndrome, rheumatic diseases, and five types of cancer [9]. Interconnections with other noncommunicable diseases, such as dental caries, are still in development [9]. These conditions may exhibit characteristic alterations in the oral environment, so the interconnections between oral health and systemic diseases have gained significant research attention in recent years [9]. The early detection of periodontitis, not solely as a method for detecting possible manifestations of other NCDs but also as a preventive intervention against potential NCD risk factors, might allow for “killing two birds with the same stone” [9].

Oral biomarkers, which include several biochemical [10,11,12,13,14,15], microbiological [16], immunological [17], and genetic indicators [18,19], have been widely reported as potential non-invasive and point-of-care tools for the early diagnosis, monitoring, and prognosis of oral diseases. However, oral biomarkers might also show additional and valuable advantages in the clinical or subclinical assessment of systemic diseases, pointing out the value of the mouth as a reflection of the systemic health status. This literature review explores the role of oral biomarkers in the assessment of well-documented systemic diseases, particularly NCDs, focusing on their diagnostic relevance and predictive capabilities. This review aims to synthesize the mechanisms through which oral fluids reflect systemic health, the current studied biomarkers, and the potential implications for clinical practice. Through a better understanding of oral biomarkers, healthcare professionals may advance toward more integrated approaches in monitoring and managing chronic conditions, embracing and encouraging interdisciplinary practice.

## 2. Oral Biomarkers: Sources

A biomarker is a measurable molecule in biological samples that indicates a biological process, condition, or disease and can be used for diagnosis, prognosis, or monitoring therapeutic responses [20]. Oral biomarkers found in oral tissues and fluids, mainly in saliva, gingival crevicular fluid, and other local sources, can provide a ready-to-go alternative in the early diagnosis and assessment of several oral and non-oral diseases and conditions.

Saliva, in the first place, is a complex and enriched fluid primarily produced by major and minor salivary glands that play roles in oral lubrication, food digestion, antimicrobial defense, and even oral homeostasis [1]. It consists of approximately 99% water and other organic and inorganic substances, including electrolytes, proteins, enzymes, immunoglobulins, and microbial constituents, as well as hormones, cytokines, and different metabolic sub-products, which can passively or actively enter the blood [21]. Therefore, saliva could be a reflection of physiological and pathological systemic conditions at the individual level and become a valuable non-invasive, easy-to-use, real-time diagnostic candidate for systemic disease assessment [22].

Gingival crevicular fluid (GCF) is a serum-derived fluid that leaks from the gingival sulcus varying from a transudate in a healthy periodontium to an exudate during periodontal inflammation, varying in both volume and composition [23]. It is composed of local periodontally derived products from resident and inflammatory cells and systemically derived molecules, including immune cells, cytokines, enzymes, and tissue-breakdown metabolites, which can provide critical insights into disease processes occurring inside and outside the periodontium. Due to its wide composition and its simple and non-invasive collection method, GCF—when tested on a paper strip for thirty seconds—is particularly useful in assessing both oral and systemic inflammatory conditions, as its constituents may reflect not only the local periodontal status but also systemic immune activity [24].

### 2.1. Oral Biomarkers in Periodontal Disease Assessment

Oral biomarkers in general, and particularly in periodontal disease assessment, can be sorted into four main categories: biochemical, immunological, genetic, and microbiological.

### 2.2. Biochemical Markers

Several biomarkers have been studied to monitor the presence and progression of oral diseases. First, matrix metalloproteinase-8 (MMP-8) can be found in saliva [15] and GCF [14] and might be used as a diagnostic tool for monitoring periodontitis and peri-implantitis [25], particularly at the individual and site levels, respectively. Matrix metalloproteinase-8 (MMP-8), also known as collagenase-2, performs as a robust oral biomarker with reliable detection in oral fluids. MMP-8’s specificity to break down the primary components of the periodontal extracellular matrix—type I and III collagens—points out/positions this enzyme as a relevant marker in periodontal pathology. Its stability in saliva and GCF further supports its role in periodontitis diagnosis, as the enzyme retains structural integrity and enzymatic function in these environments, enabling accurate and consistent measurements [26]. Notably, MMP-8 is abundant in inflammatory conditions, with elevated levels strongly associated with periodontal tissue degradation, which highlights its potential value as a biochemical indicator of the severity and activity of periodontitis [11]. Moreover, MMP-8 exists in both active and proenzyme (inactive) forms in oral fluids, providing additional insights into the enzyme’s activity and disease progression when both forms are measured [26]. MMP-8 detection methods, like enzyme-linked immunosorbent assays (ELISAs) [27], time-resolved immunofluorometric assays (IFMAs), and point-of-care salivary testing [15], offer potential non-invasive diagnostic approaches with widespread application in clinical and research settings focused on oral health and disease. However, promising research focuses on the value of the active form of MMP-8 (aMMP-8) as a chairside/point-of-care tool for assessing oral and systemic diseases [15,28].

Saliva and GCF also constitute a source of oxidative stress biomarkers and lipid peroxidation, including malondialdehyde (MDA), myeloperoxidase (MPO), nitric oxide, peroxynitrite, and 8-hydroxy-deoxyguanosine. The levels of these markers are elevated in chronic periodontitis, highlighting the contribution of oxidative stress to periodontal disease pathogenesis and progression [29,30]. Local MDA increases in GCF were associated with periodontitis severity [31], while studies that report the total oxidant and antioxidant capacity in the GCF and saliva of periodontitis patients further confirm that oxidative stress is an aggravating factor in periodontal inflammation. Indeed, increased oxidative status is correlated with a reduction in antioxidant defense, which intensifies periodontal destruction [30]. In the same way, superoxide dismutase (SOD), a critical antioxidant enzyme, plays an essential role in combating oxidative stress in chronic periodontitis patients, with increased SOD activity observed in response to inflammation and periodontal disease progression. Studies indicate that SOD levels are significantly higher in periodontitis than in healthy tissues, reflecting an adaptive response to elevated oxidative stress [32]. Apolipoprotein A-I, also present in GCF, helps in the modulation of inflammatory responses by reducing neutrophil activation and superoxide production, thus potentially protecting against tissue damage during inflammatory episodes [29]. Additionally, neutrophil defensin-1 (part of the human neutrophil peptide family), abundant in GCF during chronic periodontitis, serves as an indicator of heightened immune activity against periodontal pathogens, particularly during periodontal disease phases [33].

Vascular endothelial growth factor (VEGF) is a critical signaling protein for angiogenesis that promotes blood vessel formation. It plays essential roles in physiological processes and pathological conditions like cancer and diabetic retinopathy [34]. VEGF A levels in GCF are significantly elevated in patients with periodontal disease, particularly in inflamed and periodontally compromised sites, emerging as a potential biomarker for periodontal disease severity and progression [35]. VEGF is positively correlated with clinical periodontal parameters, like gingival index and attachment loss, which demonstrates its direct association with inflammation and tissue damage in periodontal diseases [35]. Furthermore, following periodontal therapy, VEGF levels decrease, highlighting its role in tissue remodeling [36,37,38].

### 2.3. Immunological Markers

Immunological markers, including cytokines present in the oral cavity, can reveal the immune status of organisms, providing a view into both local and systemic inflammation. Pro-inflammatory cytokines, like interleukin-1β (IL-1β) and interleukin-6 (IL-6), in GCF can be reliable indicators of periodontal disease progression due to their high sensitivity in detecting inflammatory changes in periodontal tissues [39]. Patients with chronic periodontitis presented increased levels of IL-6 and tumor necrosis factor-alpha (TNF-α) in saliva, while elevated levels of IL-6 and TNF-a in this fluid were also associated with chronic infections and autoimmune disorders [17,40]. IL-1β, IL-6, TNF-α, and other oral fluid cytokines may reflect a broader inflammatory burden, correlated with systemic diseases, as a potential valuable tool for monitoring both oral health and overall systemic inflammation [41].

### 2.4. Genetic Markers

Genetic material, including DNA and RNA, can be effectively and non-invasively collected from saliva, providing insights into genetic susceptibility to various oral and systemic diseases. Salivary microRNAs (miRNAs), especially those encapsulated in exosomes, are emerging as promising biomarkers that could help in the assessment of the disease status of conditions like periodontitis and oral cancer [42]. In this way, salivary exosomal miRNA levels, from miR-140-5p and miR-146a-5p, were correlated to periodontitis severity in some studies [43], while other miRNAs, such as miR-143-3p, have been proposed as potential non-invasive biomarkers in saliva for alternative periodontal diagnoses of chronic periodontitis patients [44]. Moreover, salivary transcriptomics has been explored for identifying individual susceptibility to chronic conditions, with preliminary research supporting its potential as a diagnostic additional tool for chronic periodontitis [45].

### 2.5. Microbiological Indicators

Periodontitis constitutes dysbiosis-triggered inflammation, commonly considered as an important source of Gram-negative anaerobic bacteria. These microorganisms might exacerbate not only local but also systemic inflammation, through the direct passage of virulence factors or bacterial products into the bloodstream and/or the indirect activation of the host’s immune response [46]. Periodontal inflammation and activities like mastication, tooth brushing, and dental procedures could facilitate bacterial translocation to serum [47].

**Figure 1:**
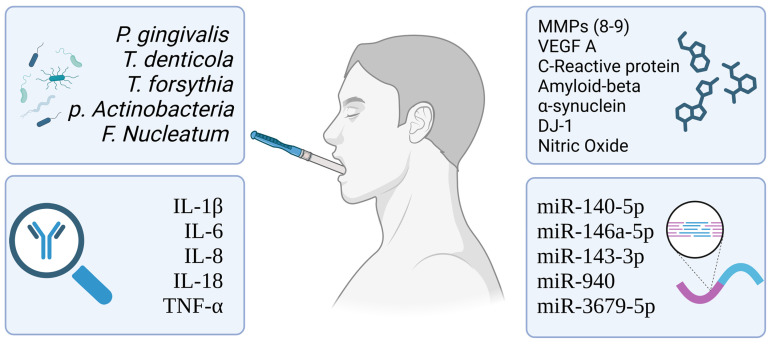
Summary of main oral biomarkers synthesized in this review. Source: BioRender.

Alterations in the oral microbiome and periodontal pathobionts, like *Porphyromonas gingivalis*, *Treponema denticola*, *Tannerella forsythia*, *Aggregatibacter actinomycetemcomitans*, and *Fusobacterium nucleatum*, among others, have been increasingly linked to local and systemic inflammation, being related to chronic and noncommunicable diseases [48,49]. In this regard, some studies have proposed that monitoring and managing oral microbiota, especially *P. gingivalis*, might offer predictive insights and preventative strategies for specific systemic diseases (Figure 1).

## 3. Systemic Diseases and Oral Biomarkers

**Figure 2:**
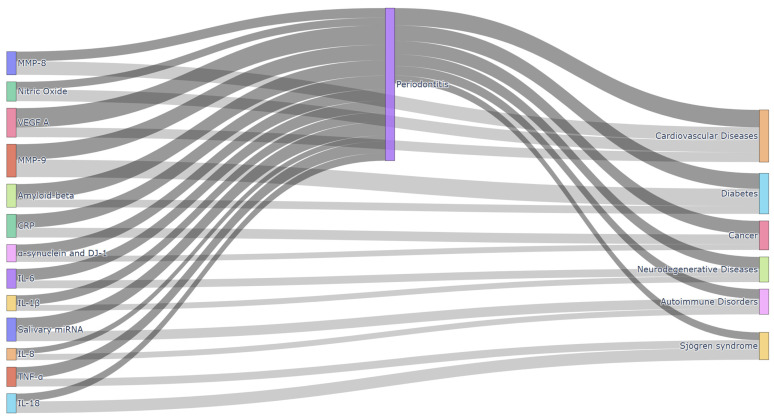
Diagram showing relationships between oral biomarkers, periodontitis, and systemic diseases. Source: Self-elaboration.

The relationship between oral biomarkers and systemic diseases is increasingly evident. Different systemic diseases exhibit unique oral biomarker profiles in oral fluids, which may appear as early indicators of disease diagnosis, progression, or severity [50] (Table 1). Systemic inflammation caused by pro-inflammatory cytokines in periodontitis like IL-6 and TNF-α contributes to cardiovascular disease, diabetes, and metabolic syndrome. Bacterial dissemination, where pathogens such as *Porphyromonas gingivalis* enter the bloodstream, is associated with atherosclerosis. Molecular mimicry by periodontal pathogens can trigger autoimmune responses, linking periodontitis to rheumatoid arthritis. Additionally, chronic infection increases oxidative stress, exacerbating conditions like neurodegenerative diseases and chronic kidney disease [51,52,53]. This section examines several systemic diseases in which oral biomarkers have emerged as a promising diagnostic tool (Figure 2).

### 3.1. Cardiovascular Diseases

Numerous studies indicate that periodontal pathogens and inflammatory biomarkers present in the oral cavity are closely linked to cardiovascular diseases (CVDs). For example, *Porphyromonas gingivalis*, a pathobiont associated with periodontal disease, is implicated in increasing atherosclerosis risk [54,55]. This pathogen has been detected in atherosclerotic plaques and promotes inflammation in vascular tissues by inducing the expression of pro-inflammatory cytokines and oxidative stress in endothelial cells [56]. Additionally, *P. gingivalis* interacts with dendritic cells, where bacteria can destabilize atherosclerotic plaques and contribute to coronary artery disease [57]. Chronic infection with *Tannerella forsythia* elevates serum lipoprotein levels. This suggests that bacterial infection may directly alter lipid metabolism, with the resulting increase in lipoproteins contributing to the inflammatory process in the aorta. [58].

The measurement of oral inflammatory biomarkers could also help in monitoring cardiovascular diseases as a screening tool. Whereas C-reactive protein (CRP) is a validated marker of cardiovascular disease in serum [50], elevated levels of salivary CRP are also associated with CVD incidence, and using CRP has been proposed as a non-invasive and accessible method in the future assessment of cardiovascular risk and systemic inflammation [59,60]. A study by our group proved that CRP levels in GCF were statistically elevated in women with periodontal diseases and correlated with CRP detected in serum in all study groups (periodontitis, gingivitis, and controls) [61]. These findings support that GCF CRP might be a potentially simple and non-invasive aid for cardiovascular risk assessment and other inflammatory-driven health conditions [62]. Interestingly, serum CRP levels were also found to be elevated in periodontitis patients [63], while periodontal disease therapy reduced systemic inflammatory markers of atherosclerosis progression, like CRP and interleukins, highlighting the potential systemic benefits of managing oral infections [64].

High levels of MMP-8 and MPO in periodontitis patients have been associated with waist circumference, blood pressure, and triglycerides, indicating their potential utility for monitoring cardiovascular and glycemic risk in patients with periodontitis [65], and they have been used to evaluate pharmacologic response to therapeutic intervention [66]. Other salivary biomarkers, such as Myoglobin (MYO), Cardiac troponin I (cTnI), Creatine phosphokinase MB (CK-MB), brain natriuretic peptide (NT-proBNP), exosomal miRNA, MMP-9 [67], and the tissue inhibitor of MMP-8 (TIMP-1), leukotriene B4, have been well reported on and reviewed in the last decade as indicators of CVD [68].

#### Cardiorenal Syndrome: Association Between Cardiovascular Diseases and Chronic Kidney Disease

Cardiorenal syndrome encompasses a spectrum of disorders involving both the heart and kidneys in which acute or chronic dysfunction in one organ may induce acute or chronic dysfunction in another organ [69]. Classical biomarkers for the assessment of kidney function include serum creatinine, albuminuria, and cystatin C, as well as urine output and estimated glomerular filtration rate [70]. Non-invasive salivary assessments of creatinine, cystatin C, and urea make it feasible to conduct the frequent monitoring of kidney function in point-of-care settings, as well as in nonclinical care settings such as at home [71]. Oral biomarkers have shown correlations with serum biomarkers, for example, trimethylamine N-oxide, indicating that saliva can be successfully used in the non-invasive monitoring of renal failure in chronic kidney disease (CKD) by measuring salivary TMAO concentrations [72]. Salivary creatinine levels showed a sensitivity of 93.3% and 87.1% for specificity, while salivary urea levels showed a sensitivity of 87.5% and 83.2% for specificity for the determination of the uremic state in patients with CKD [73,74]. Oral biomarkers have been positioned as a novel and feasible alternative for monitoring CKD, especially in low-resource settings, where blood serum screening is unavailable [75,76]. Monitoring the status of CKD would contribute to reducing the complications associated with CVD, reducing the morbidity related to both diseases [77].

The microbiome is also altered in patients with CKD, showing higher levels of Lautropia and Pseudomonas and decreased levels of Actinomyces, Prevotella, Prevotella 7, Trichococcus [78,79], and Neisseria [80]. However, salivary biomarkers for CKD are still in development, and CKD’s relationship with periodontal disease is still unclear [81].

### 3.2. Type 2 Diabetes Mellitus

Salivary biomarkers, particularly glucose levels, IL-6, TNF-α, CRP, and specific salivary biomolecules, such as adiponectin, leptin, and 1,5-Anhydroglucitol, correlate strongly with T2DM, offering an accessible method for assessing and monitoring the condition [82]. Elevated glucose levels in saliva correlates with serum hyperglycemia in diabetic patients, enabling potential non-invasive straightforward monitoring. Additionally, the levels of inflammatory biomarkers, including IL-6 and TNF-α, were found to be significantly higher in the saliva of diabetic patients, reflecting the tissue damage and systemic inflammation associated with diabetes [83]. Indeed, the levels of TNF-α and IL-6 in saliva have been associated with the severity of diabetic retinopathy [84]. Additionally, the salivary levels of Alpha-2 Macroglobulin are correlated to the serum levels of glycate hemoglobin A1c [85]. Furthermore, periodontitis and T2DM are correlated with nuclear abnormalities, particularly in jugal epithelial cells, as well as salivary reduced glutathione and uric acid levels [86]. Salivary AGEs and aMMP-8 are also associated with uncontrolled T2DM in patients with periodontitis [87]. These saliva biomarkers, in addition to C-reactive protein (CRP), support the use of this fluid as a method to aid in the evaluation of systemic inflammation and glycemic control in diabetes patients [83].

Additionally, the oral microbiome of patients with T2DM has revealed interesting findings. At the phylum level, the salivary microbiota of T2DM patients and healthy controls was dominated by phylum Firmicutes, followed by Bacteroidetes, Proteobacteria, Fusobacteria, and Actinobacteria, accounting for approximately 95% of the total bacteria [88].

### 3.3. Autoimmune Disorders

Autoimmune diseases, such as rheumatoid arthritis (RA) and Sjögren’s syndrome, exhibit unique oral manifestations and specific biomarker profiles that could aid in diagnosis and disease assessment. For instance, Sjögren’s syndrome, a condition that targets salivary glands, leads to an altered composition and reduced saliva production. Characteristic autoantibodies, such as anti-Ro and anti-La, act as key diagnostic indicators [89]. Additionally, elevated levels of cytokines, including IL-18, have been observed in the saliva and serum of patients with Sjögren’s syndrome, indicating immune activation within the salivary glands and providing a potential marker of disease severity [90]. In addition, the salivary levels of soluble siglec-5 correlate with the grade of hyposalivation and ocular surface damage in patients with this syndrome [91].

Similarly, in RA, increased levels of pro-inflammatory cytokines, like IL-6 and TNF-α in saliva, mirror systemic immune activity, which may turn these markers into useful evaluation tools in the assessment of inflammation levels and disease progression [92]. RA patients exhibit significantly higher salivary levels of IL-6 and TNF-α, which reflect serum inflammation levels and correlate with disease activity scores, emerging as a method for monitoring RA progression [93]. Additionally, salivary IL-1β levels have been observed to be higher in RA patients, especially in those without anti-TNF-α therapy, suggesting that salivary cytokine levels may also be associated with the response to RA therapies [94]. Further studies should confirm whether RA patients with elevated salivary IL-17A and IL-8 levels experience greater periodontal inflammation, reinforcing the interplay between oral and systemic inflammation in RA [95]. Additionally, genome-wide association studies (GWASs) have identified multiple risk alleles related to nuclear-factor (NF)-κB, which thus confer a genetic predisposition to developing RA or other autoimmune disorders [96].

In the same way, studies have found that *P. gingivalis* infection was correlated with elevated disease activity in rheumatoid arthritis, potentially mediated by bacteria citrullination, an immune-modulating process central to RA pathogenesis [97]. Additionally, *P. gingivalis* has been shown to worsen gut microbiota dysbiosis and arthritis severity in animal models, highlighting its role in the promotion of systemic inflammation beyond the oral cavity [98].

### 3.4. Cancer

Emerging research on salivary biomarkers reveals that specific genetic mutations, RNA, and protein markers in saliva are associated with cancers, particularly head and neck cancers [99]. Indeed, from 2010 to 2024, 35 unique analytes were identified [100]. Reported biomarkers include TNF-α, IL-1β, IL-6, IL-8, lactate dehydrogenase (LDH), and MMP-9, with high sensitivity and specificity values in almost all studies, and TNF-α, IL-1β, IL-6 IL-8, LDH, and MMP-9 were reported as the most promising salivary constituents. Regarding oral squamous cell carcinoma, Chemerin and MMP-9 displayed the highest sensitivity (0.94); for head and neck squamous cell carcinoma, Actin, IL-1β, and IL-8 showed the highest accuracy, with an area under the curve of 0.79, 0.75, and 0.70, respectively [101]. Other biomarkers, such as NF-κB, have shown potential regarding their role in block cell proliferation [102]. Mutations in tumor suppressor genes like *p16*, *RASSF1A*, and *TIMP3*, detected through DNA methylation in saliva, have shown high specificity for identifying the early stages of oral and oropharyngeal cancers [103]. Furthermore, aberrant DNA methylation patterns in salivary samples can be used to predict cancer recurrence in head and neck squamous cell carcinoma, providing a valuable non-invasive surveillance tool for these malignancies [104]. In addition, specific salivary cytokines, such as IL-8, have been studied for their potential to reflect tumor presence and progression, demonstrating diagnostic accuracy in detecting head and neck cancers [105]. Additionally, salivary transferrin is a potential candidate as an early detection biomarker and a prognostic marker for oral cancer, allowing for the development of diagnostic tests [106].

In other aspects, it has been demonstrated in a recent meta-analysis that salivary biomarkers (proteo- and metabolomic) had a pooled accuracy of 0.8 in detecting patients with breast cancer compared to healthy controls [107]. Evidence also suggests that salivary miR-940 and miR-3679-5p are reliable markers for pancreatic cancer and that miR140-5p and miR301a are promising molecules for the salivary diagnosis of gastric cancer [108]. In addition, changes in the concentration of salivary VEGF A and related cytokines in saliva indicate different molecular biological subtypes of breast cancer depending on the stage of the disease, differentiation, proliferation, and metastasis to the lymph nodes [109].

### 3.5. Neurodegenerative Disorders

Recent studies have identified promising oral biomarkers for diagnosing and monitoring neurodegenerative diseases, which offer a non-invasive alternative to traditional methods [110]. Recent evidence has shown that saliva is the source of β-amyloid, tau protein, α-synuclein, DJ-1, and Huntington protein that display reliability and validity as the biomarkers of neurodegenerative diseases [110]. Saliva has been shown to contain key biomarkers associated with Alzheimer’s disease, such as amyloid β_1-42_, tau proteins, and lactoferrin, and some studies have already proposed their potential for the early detection and monitoring of disease progression [111,112,113]. Similarly, α-synuclein and protein deglycase (DJ-1) have been described as promising diagnostic biomarkers for Parkinson’s disease [114,115]. Additionally, oral microbiota changes have been observed in Parkinson’s disease, with a shift in salivary and subgingival microbial composition (particularly Gram-negative bacteria), potentially applicable as an early diagnostic tool for the disease [116]. Collectively, these oral biomarkers are progressively being considered and recognized for their potential to revolutionize early diagnosis and personalized treatment approaches in neurodegenerative disorders.

## 4. Challenges, Limitations, and Future Directions

Despite their potential, the integration of oral biomarkers into routine clinical practice faces several challenges and limitations. These obstacles range from technical and analytical issues to broader considerations, related to measurement variability and clinical applicability. Addressing these challenges is essential for advancing the clinical utility of oral biomarkers and ensuring their reliability in systemic disease assessment.

### 4.1. Technical and Analytical Limitations

Emerging research on oral biomarkers has underscored significant challenges regarding their sensitivity and specificity, particularly in clinical applications. Several studies have highlighted the impacts of pre-analytical factors, such as sample collection methods, storage conditions, and assay technologies, on the accuracy and reliability of oral biomarkers. For example, variations in saliva collection techniques and discrepancies in sample handling could lead to inconsistent results between research settings and real-world clinical environments. One study found that the results of an assessment of salivary biomarkers for oral cancer detection were significantly affected by storage conditions and collection methods, underscoring the need for standardized protocols [117]. Similarly, research examining the impact of storage conditions on saliva samples revealed that improper handling could lead to substantial variation in biomarker levels, thereby affecting diagnostic accuracy [118]. Furthermore, variability in laboratory processing techniques was noted as a critical barrier to the clinical implementation of oral biomarkers, with some assays yielding unreliable results in diverse patient populations [119]. These findings point out the urgent need for uniform methodologies in the pre-analytical and analytical phases of biomarker testing. Standardization remains a key requirement for the successful implementation of oral biomarker testing. Future research should focus on establishing universally accepted protocols for sample collection, handling, and analysis. Standardized protocols will minimize variability and ensure consistency, facilitating the integration of oral biomarker testing into clinical practice. This may involve the creation of guidelines for the handling of saliva and oral fluid samples, as well as developing cost-effective and accessible diagnostic assays.

### 4.2. Inter-Individual Variability

Oral biomarker levels can be influenced by multiple factors such as age, gender, genetics, lifestyle, and oral hygiene, and this variability may complicate diverse test interpretations. One study emphasizes that genetic factors control the plaque microbiome, which could also impact the concentration of oral biomarkers [120], suggesting that microbiological markers might not become a uniform clinical standard [88]. Moreover, tobacco use and diet (notably high-carbohydrate foods) are associated with changes in the microbial and oral health status, affecting biomarker readings [121]. Additionally, systemic diseases like diabetes mellitus, often linked to periodontal conditions, can further alter biomarker levels, leading to potential misinterpretation in disease risk assessment [122]. It is crucial to account for these factors when developing diagnostic thresholds to improve the accuracy of biomarker-based screenings in oral health.

### 4.3. Integration into Clinical Practice

Although the non-invasive nature of oral biomarker testing is advantageous, some barriers must be removed before it can be adopted in routine clinical practice. These barriers include the need for specialized equipment, staff training, and the associated costs of implementing new testing protocols. Additionally, many oral biomarkers are still in research and require further validation through large-scale clinical trials to establish their efficacy and clinical relevance. Without this validation, clinicians may be hesitant to rely on oral biomarkers as definitive indicators of systemic health. While numerous biomarkers have demonstrated valuable potential in preliminary research, large-scale, multicenter validation studies are essential to confirm their diagnostic, monitoring, and prognostic accuracy across diverse populations [121,123]. Such studies should aim to establish standardized reference values, validate biomarkers for different systemic diseases, and evaluate their reproducibility in real-world clinical settings. Establishing these biomarkers as reliable tools requires robust evidence gathered through large and diverse sample sets. In addition, some authors suggest that it is unlikely that a single diagnostic biomarker will suffice for universal translation into clinical practice. Therefore, optimizing oral biomarkers, especially the combination of them in “risk profiles”, needs to be conducted as well [124].

Additionally, given the interconnections between oral and systemic health, interdisciplinary research efforts involving dental, medical, biochemical, and bioinformatics experts are essential [125]. Collaborative research will enable a more comprehensive understanding of how oral biomarkers reflect systemic conditions and promote innovations in integrated healthcare. Such efforts can also help to identify novel biomarkers with higher specificity and sensitivity and explore advanced analytical methods, such as machine learning, to improve biomarker discovery and interpretation [125]. In addition to commonly studied biomarkers, researchers should investigate emerging indicators, including novel genetic, proteomic, and metabolomic markers, to expand the range of detectable conditions [126]. Advanced technologies such as next-generation sequencing and mass spectrometry may facilitate the identification of these new biomarkers.

Importantly, the development of portable and affordable point-of-care devices for oral biomarker analysis will significantly enhance accessibility to this technology [15]. Future research should focus on creating user-friendly diagnostic devices that provide rapid and accurate results at the point of care, facilitating early detection and ongoing monitoring. These devices could also support remote healthcare, enabling patients to monitor their health from home and potentially reducing healthcare disparities.

### 4.4. Regulatory and Economical Concerns

**Figure 3:**
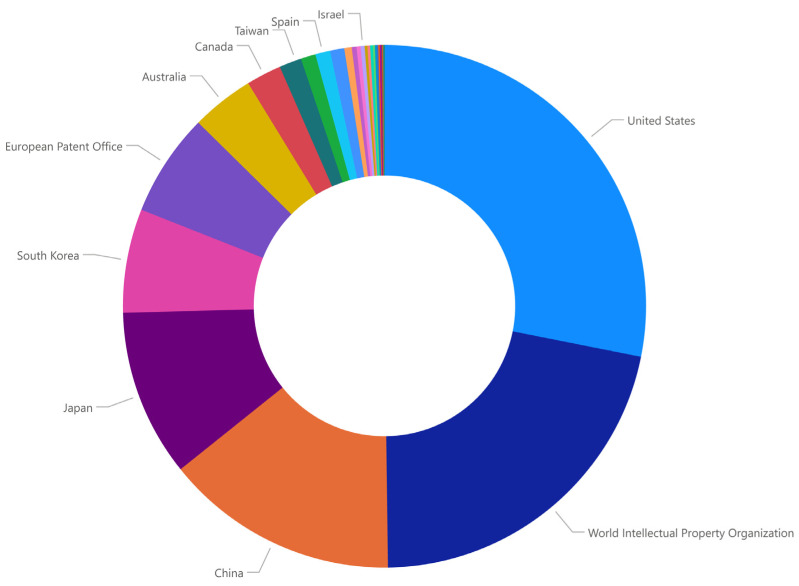
Percentage of patents of oral biomarkers for assessment of noncommunicable diseases by country (or organization). Source: data from Google Patents after searching for “oral biomarkers AND noncommunicable diseases” on 26 December 2024 (10,278 results). Source: Self-elaboration.

Regulatory challenges surrounding the clinical adoption of oral biomarkers necessitate alignment with international standards, such as those established by the FDA and EMA, to ensure their recognition as reliable diagnostic tools. Additionally, the registration of patents in international organizations is mandatory, as the research involved in the assessment of oral biomarkers, especially point-of-care research, implies, in most cases, the development of an instrument. Currently, there are 10,278 published patents for oral biomarkers for the assessment of NCDs in Google Patents, with registries being concentrated in the United States (28%), the World Intellectual Property Organization (~22%), China (14%), Japan (10%), South Korea (6%), and the European Patent Office (6%) (Figure 3). Clear guidelines must be developed for evaluating biomarkers’ sensitivity, specificity, reproducibility, and clinical relevance to facilitate their validation and approval. For example, compliance with current standards, such as the guidance from the WHO on diagnostic tools using the WHO REASSURED criteria, could optimize efforts in the research of oral biomarkers [127] (Table 2). Additionally, ethical concerns related to patient data privacy are paramount, particularly with the integration of machine learning algorithms and point-of-care diagnostic devices. In this sense, robust data protection measures are essential to maintain trust and comply with regulations such as GDPR and HIPAA, particularly for data storage and monitoring, particularly when developing big-data repositories for world biomarker data.

In addition, there may be economic limitations of current and other oral biomarkers, as the costs of development, validation, and implementation may limit their scalability in low-resource settings. Many challenges exist beyond price and availability for the current tools included in the Package of Essential Noncommunicable Disease Interventions (PEN) for cardiovascular disease, diabetes, and chronic respiratory diseases. These include temperature stability, adaptability to various settings (e.g., at high altitude), need for training to perform and interpret the test, the need for maintenance and calibration, and so on [127]. The simplification of oral biomarker assessment and implementation, particularly in the analysis of samples, is a challenge in future developments, as the main poles of the development of this research topic are high-income countries and organizations. Additionally, pilot programs and real-world initiatives demonstrating the successful integration of biomarkers into clinical workflows are vital for establishing best practices and building a regulatory pathway that accelerates their translation into routine care.

Addressing these challenges requires innovative solutions, including public–private partnerships and cost-sharing models, to reduce financial barriers. Integration into broader healthcare systems is also critical; biomarkers must be incorporated into existing healthcare infrastructure, such as electronic health records (EHRs), to streamline diagnostics and support preventive healthcare strategies [128]. By enabling early detection and population health management, biomarkers could significantly enhance systemic health outcomes.

**Figure 4:**
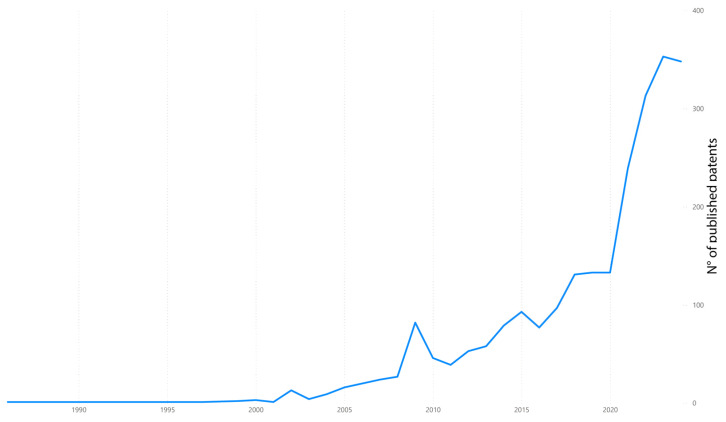
The evolution of the number of patents of oral biomarkers for the assessment of noncommunicable diseases by year. Source: data from Google Patents after searching for “oral biomarkers AND noncommunicable diseases” on 26 December 2024 (10,278 results). Source: Self-elaboration.

In summary, while oral biomarkers hold significant promise, addressing these challenges is essential to advance their clinical applicability. Oral biomarkers have shown promise both as early indicators of disease and as predictive factors. Some biomarkers may appear before the clinical manifestation of systemic diseases, offering a valuable opportunity for early detection and intervention. As shown in this review, inflammatory markers in saliva are associated with cardiovascular and metabolic conditions before overt symptoms develop. However, the extent to which they reliably predict disease progression or risk remains an area of active research. Validation through longitudinal studies is crucial to establish their predictive capabilities and determine their role in risk stratification and monitoring. This topic is a source of exponential research development in the last decade, with over a hundred patents published every year (Figure 4). By overcoming technical limitations, reducing variability, and establishing standardized protocols, oral biomarkers can become a valuable component of integrated healthcare approaches.

## 5. Conclusions

Oral biomarkers represent a transformative approach to understanding and managing systemic diseases, offering a non-invasive and accessible window into an individual’s overall health. The body of evidence linking oral biomarkers with conditions such as cardiovascular disease, diabetes, autoimmune disorders, and cancers continues to grow, highlighting the diagnostic and predictive potential of these biomarkers in clinical settings. Compared to conventional serum markers, oral biomarkers offer advantages in convenience and patient compliance, making them particularly valuable for both dentists and general practitioners. Their ease of collection supports their integration into routine dental and medical check-ups, broadening their applicability in preventive care and the early detection of systemic diseases. Looking ahead, oral biomarkers have the potential to become essential components of personalized medicine, bridging oral and systemic health management.

Despite the promising potential of oral biomarkers, several challenges remain to be solved, including standardization issues, inter-individual variability, and the need for larger validation-focused studies. Addressing these challenges will be essential to fully develop the clinical utility of oral biomarkers and integrate them into routine healthcare practices. Continued interdisciplinary research and technological advancements will likely propel this field forward, paving the way for innovations in early diagnosis, disease monitoring, and personalized treatment strategies.

Ultimately, the integration of oral biomarkers into healthcare systems could mark a significant step toward preventive and patient-centered care, helping clinicians in the early detection, monitoring, and management of systemic diseases through simple and non-invasive methods that may lead to improved patient outcomes and a more holistic approach to health.

## Figures and Tables

**Table 1:** Summary of main oral biomarkers, their associated diseases, and clinical uses.

Biomarker	Source	Associated Diseases	Potential Clinical Use
MMP-8	Saliva, GCF	Periodontitis, Cardiovascular Diseases, Diabetes	Non-invasive diagnosis, Cardiovascular risk assessment
MMP-9	Saliva	Cardiovascular disease, Cancer	Early diagnosis
CRP	Saliva, GCF	Cardiovascular Diseases, Diabetes	Cardiovascular risk assessment, Disease progression assessment
IL-6	Saliva, GCF	Periodontitis, Diabetes, Rheumatoid Arthritis, Cancer	Inflammation monitoring, Disease progression assessment
IL-8	Saliva	Cancer	Early diagnossis
IL-18	Saliva	Sjögren syndrome	Disease severity assessment
VEGF A	Saliva, GCF	Periodontitis, Cancer	Disease progression assessment
Amyloid-beta	Saliva	Alzheimer’s disease	Early diagnosis
α-synuclein and DJ-1	Saliva	Parkinson’s disease	Early diagnosis
IL-1β	Saliva, GCF	Periodontitis, Rheumatoid Arthritis, Cancer	Systemic inflammation monitoring, Early diagnosis
Salivary miRNA (miR-140-5p, miR-146a-5p, miR-143-3p, miR-940 and miR-3679-5p)	Saliva	Cancer, Cardiovascular Diseases	Early diagnosis
TNF-α	Saliva	Periodontitis, Type 2 Diabetes Mellitus, Rheumatoid Arthritis	Systemic inflammation monitoring, Disease progression and severity assessment
Nitric Oxide	Saliva	Periodontitis, Neurodegenerative Diseases	Oxidative stress evaluation

Abbreviations: CRP: C-reactive protein; GCF: gingival crevicular fluid; IL-1β: interleukin-1 beta; IL-6: interleukin-6; IL-8: interleukin-8; IL-18: interleukin-18; miRNA: microRNA; MMP-8: matrix metalloproteinase-8; MMP-9: matrix metalloproteinase-9; TNF-α: tumoral necrosis factor- α; VEGF: vascular endothelial growth factor.

**Table 2:** WHO REASSURED criteria for diagnostic test included in WHO PEN package.

Criteria	Description
Real-time connectivity	Tests are connected, and/or a reader or mobile phone is used to power the reaction and/or read test results to provide required data to clinicians and users.
Ease of specimen collection	Tests should be designed for use with non-invasive specimens.
Affordable	Tests are affordable to end-users and the health system.
Sensitive	Avoid false negatives.
Specific	Avoid false positives.
User-friendly	The procedure of testing is simple—it can be performed in a few steps, requiring minimal training.
Rapid and robust	The results are available to ensure the treatment of patients at their first visit (typically, this means that results are obtained within 15 min to 2 h), and the test can survive the supply chain without requiring additional transport and storage conditions such as refrigeration.
Equipment-free and environmentally friendly	Ideally, the test does not require any special equipment or can be operated using very simple devices that use solar or battery power. Completed tests are easy to dispose of and manufactured from recyclable materials.
Deliverable to end-users	Accessible to those who need the tests the most.
